# Efficacy and safety of fluticasone furoate nasal spray in adult and adolescent subjects with uncomplicated acute rhinosinusitis

**DOI:** 10.1186/1710-1492-7-S2-A41

**Published:** 2011-11-14

**Authors:** Paul Keith, Andrzej Dymek, Oliver Pfaar, Wytske Fokkens, Nazli Topors, Wei Wu, Suyong Yun Kirby, Laurie A Lee

**Affiliations:** 1 McMaster University, Hamilton, ON, Canada; 2 Centrum Medyczne Lucyna Andrzej Dymek NZOZ S.C., Poland; 3 University Hospital Mannheim, Wiesbaden, Germany; 4 Academisch Medisch Centrum, Amsterdam, The Netherlands; 5 GlaxoSmithKline, Mississauga, ON, Canada; 6 GlaxoSmithKline, Research Triangle Park, NC, USA

## Background

Uncomplicated acute rhinosinusitis (ARS) is usually a self-limiting inflammatory condition often treated with antibiotics. This study evaluated an alternative treatment for symptomatic relief of uncomplicated ARS, fluticasone furoate nasal spray (FFNS).

## Methods

This randomized, double-blind, placebo-controlled, parallel-group, multicenter, 2-week treatment study evaluated FFNS 110 mcg once daily, twice daily vs. placebo in adults/adolescents with uncomplicated ARS. Eligibility criteria reflected a clinical diagnosis and eliminated confounding conditions like common cold, symptomatic allergic rhinitis (AR), and other sinonasal conditions. Subjects with daily major symptom score (MSS; a composite score of 3 symptoms [nasal congestion/stuffiness, sinus headache/pressure or facial pain/pressure, and postnasal drip on a 0-3 scale]) >4.5 at baseline were randomized.

## Results

The study demonstrated a statistically significant reduction in daily MSS by both FFNS doses compared to placebo (LS mean differences vs. placebo of -0.357 [p=0.014] and -0.386 [p=0.008] for BID and QD, respectively). The differences in median time to symptom improvement between placebo (8 days) and each FFNS dose (7 days) were not statistically significant. There were no treatment differences in antibiotic use due to the development of possible fulminant bacterial rhinosinusitis (3% in each group). The safety profile of FFNS was similar to placebo.

## Conclusion

FFNS reduced symptoms of uncomplicated ARS compared to placebo and was well tolerated, providing support for withholding antibiotics in selected patients. (FFR113203/ GSK funded)

